# Novel Arenavirus Isolates from Namaqua Rock Mice, Namibia, Southern Africa

**DOI:** 10.3201/eid2107.141341

**Published:** 2015-07

**Authors:** Peter T. Witkowski, René Kallies, Julia Hoveka, Brita Auste, Ndapewa L. Ithete, Katarína Šoltys, Tomáš Szemes, Christian Drosten, Wolfgang Preiser, Boris Klempa, John K.E. Mfune, Detlev H. Kruger

**Affiliations:** Charité Medical School, Berlin, Germany (P.T. Witkowski, B. Auste, B. Klempa, D.H. Kruger);; Helmholtz Centre for Environmental Research–UFZ, Leipzig, Germany (R. Kallies);; University of Bonn Medical Center, Bonn, Germany (R. Kallies, C. Drosten);; University of Namibia, Windhoek, Namibia (J. Hoveka, J.K.E. Mfune);; Stellenbosch University and National Health Laboratory Services Tygerberg, Cape Town, South Africa (N.L. Ithete, W. Preiser);; Comenius University, Bratislava, Slovakia (K. Šoltys, T. Szemes);; Slovak Academy of Sciences, Bratislava (B. Klempa)

**Keywords:** arenavirus, Africa, Namibia, zoonotic infectious diseases, emerging diseases, zoonoses, viruses, rock mice

## Abstract

Arenaviruses are feared as agents that cause viral hemorrhagic fevers. We report the identification, isolation, and genetic characterization of 2 novel arenaviruses from Namaqua rock mice in Namibia. These findings extend knowledge of the distribution and diversity of arenaviruses in Africa.

Arenaviruses are known to cause severe hemorrhagic fevers across the globe with case fatality rates up to 30% (*1*). The viruses possess a bisegmented, single-stranded RNA genome with ambisense coding strategy consisting of a small segment coding for the nucleoprotein and glycoprotein and a large (L) segment coding for the RNA-dependent RNA polymerase and matrix protein.

In Africa, Lassa virus (LASV) and Lujo virus are the only known members of the family *Arenaviridae* that cause human disease (*2*,*3*); however, evidence for lymphocytic choriomeningitis virus, another *Arenaviridae* sp., was recently reported in Gabon (*4*). Several other arenaviruses of unknown pathogenic potential have also been found in Africa: Gbagroube, Kodoko, and Menekre viruses from western Africa (*5*,*6*); Ippy (IPPYV) and Mobala viruses from central Africa; Mopeia, Morogoro, Luna, and Lunk viruses from eastern Africa; and Merino Walk virus (MWV) from southern Africa (*7**,**8*). All of these viruses are carried by rodents of the family *Muridae*.

Until now, no molecular detection of arenaviruses has been reported from Namibia. A study in 1991 described a low seroprevalence (0.8%) for LASV antibodies in humans in northern Namibia (*9*). Because of lack of data about arenavirus occurrence and effects in southwestern Africa, we conducted a study of small mammals from Namibia to detect infection with arenaviruses.

## The Study

**Figure 1:**
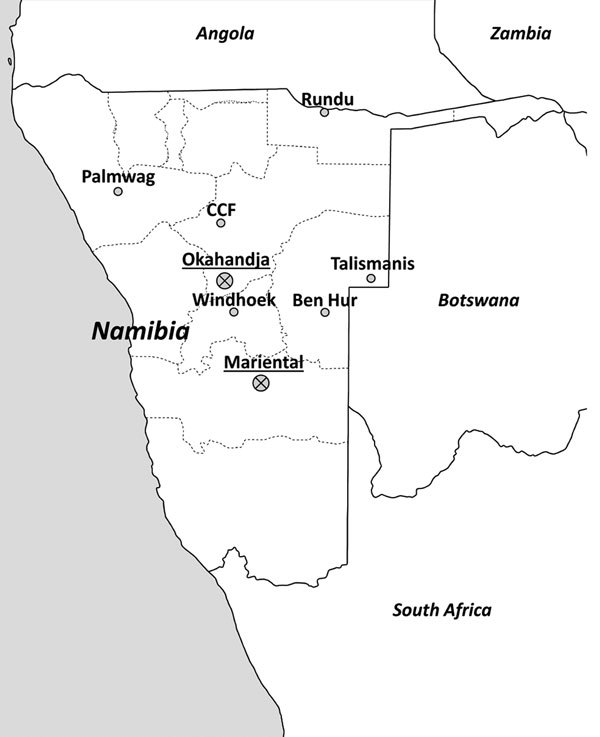
Screening for arenaviruses in Namibia. Trapping locations (named according to the nearest urban settlement) of small mammals. Sites where samples positive for new arenaviruses were found are marked by a crossed circle and underlined locality names. Geographic positioning system coordinates of the trapping sites: Ben Hur, 22°87.26′S, 19°21.10′E; Cheetah Conservation Fund (CCF), 16°39.0′E, 20°28.12′S; Mariental, 24°62.08′S, 17°95.93′E; Okahandja, (21°98.33′S, 16°91.32′E); Palmwag, 19°53.23′S, 13°56.35′E; Rundu, 17°56.645′S, 20°05.109′E; Talismanis, 21°84.30′S, 20°73.91′E; Windhoek, 22°49.93′S, 17°34.76′E.

During 2010–2012, animal trapping was performed in 8 areas in central and northern Namibia (Figure 1), and samples from 812 rodents and shrews were obtained (Table 1). The animals were dissected in the field and stored individually in a field freezer at −20°C and later at −80°C. For primary arenavirus screening, lung sections of all animals were homogenized, and RNA was extracted and reversely transcribed by using random hexamer primers. Screening was performed by arenavirus genus-specific reverse transcription PCR (RT-PCR) (*10*) to detect the L genomic segment. From samples testing positive by arenavirus PCR, frozen lung tissue aliquots were homogenized and added to confluent Vero-E6 cells (ATCC CRL-1586; American Type Culture Collection, Manassas, VA, USA) for virus isolation.

**Table 1:** Small mammals captured in Namibia during 2010–2012 and tested for arenaviruses*

Mammal species	Common name	Localities of capture†	No. positive/no. tested
*Aethomys chrysophilus *	Red veld rat	Be, CCF, Ok, Pa, Ta	0/64
*Micaelamys namaquensis*	Namaqua rock mouse	CCF, Ma, Ok, Pa, Ru	4/266
*Crocidura fuscomurina*	Bicolored musk shrew	CCF, Pa, Ru	0/4
*Crocidura hirta*	Lesser red musk shrew	Ma	0/5
*Dendromus melanotis*	Gray climbing mouse	Ta	0/1
*Elephantulus intufi*	Bushveld sengi	CCF, Ma, Ok	0/14
*Gerbilliscus *spp.	Gerbil	Wi	0/6
*Gerbilliscus leucogaster*	Bushveld gerbil	Be, CCF, Ma, Ok, Pa, Ru, Ta	0/228
*Gerbillurus paeba*	Hairy-footed gerbil	Be	0/3
*Gerbillurus setzeri*	Namib brush-tailed gerbil	Be	0/1
*Lemniscomys rosalia*	Single-striped grass mouse	Be	0/2
*Mastomys *spp.	Multimammate mouse	Be, CCF, Ma, Ok, Pa, Ru, Ta	0/114
*Mus indutus*	Desert pygmy mouse	Ma, Pa	0/5
*Petromyscus collinus*	Pygmy rock mouse	Pa	0/3
*Rhabdomys pumilio*	Four-striped grass mouse	CCF, Ma, Pa, Ok, Wi	0/73
*Saccostomus campestris*	Pouched mouse	Be, CCF, Ok, Pa, Ru	0/17
*Thallomys paedulcus*	Acacia rat	Pa	0/4
*u.u. Soricidae*	Shrew	Wi	0/2
Total			4/812

*Morphologic species identification of the arenavirus positive rodent samples was confirmed by sequencing of partial mitochondrial cytochrome b gene (GenBank accession nos.: N27, KP752173; N73, KP752175; N80, KP752176; N85, KP752174). †Abbreviations and sampling dates for trapping localities: Be, Ben Hur (11/2011); CCF, Cheetah Conservation Fund (02/2011); Ok, Okahandja (06/2012), Pa, Palmwag (09/2010), Ta, Talismanis (12/2011), Ma, Mariental (06/2012), Ru, Rundu (01/2011),Wi, Windhoek (09/2010 and 01/2012).

For genome sequencing, pellets from ultracentrifuged supernatant of infected cell cultures were lysed, and total RNA was purified. RNA was then subjected to random-primed RT-PCR as described (*11*). Next-generation sequencing was performed by using a 454 Genome Sequencer Junior (Roche, Indianapolis, IN, USA), and results were aligned against the virus database by using blastn and blastx algorithms (http://blast.ncbi.nlm.nih.gov/Blast.cgi). Sequencing results matching arenavirus sequences were mapped to the LASV strain Josiah (GenBank accession no. AY628203). Because of low coverage for N27, an additional MiSeq (Illumina, San Diego, CA, USA) run was performed. De novo assembly of the data was performed with Geneious software (Biomatters, Auckland, New Zealand) (*12*). Sequence gaps or regions with low coverage were verified by Sanger sequencing (Applied Biosystems, Foster City, CA, USA). Genome segment outermost noncoding termini were sequenced after linkage by T4-RNA-Ligase (New England Biolabs, Ipswich, MA, USA) and RT-PCR amplification.

**Figure 2:**
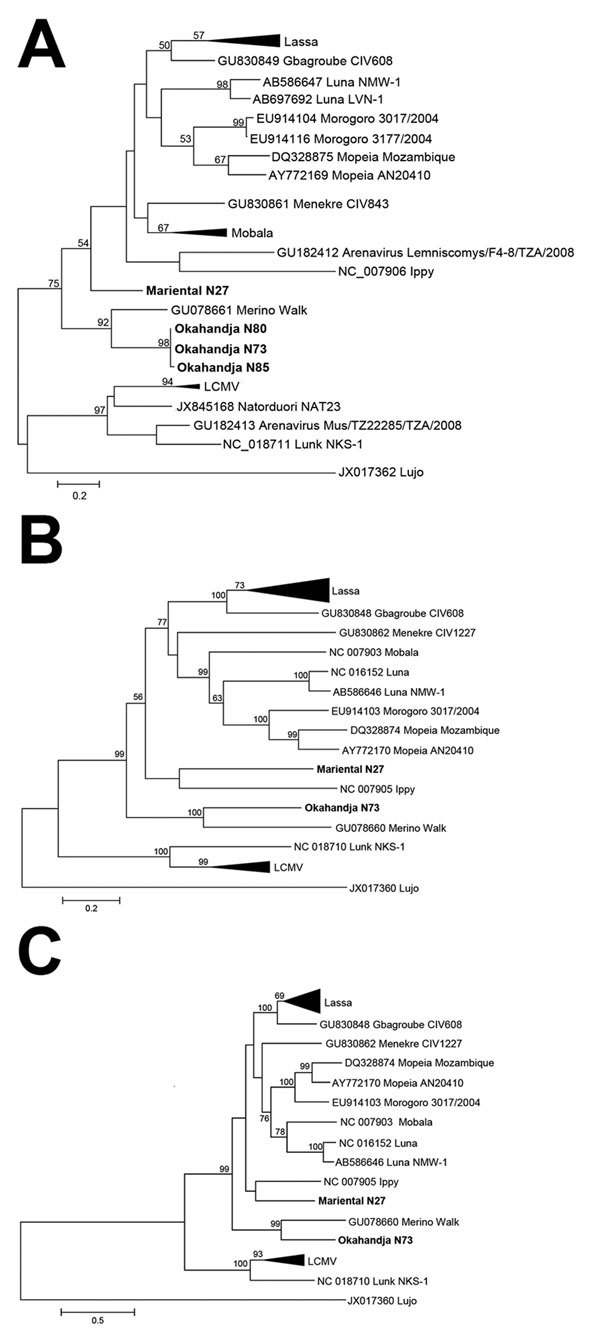
Phylogenetic analysis of Okahandja and Mariental viruses performed with maximum-likelihood method. A) Phylogenetic analysis of partial L segment sequence (338 nt) of Okahandja and Mariental viruses obtained from reverse transcription PCR screening and performed with MEGA 6.0 (*13*) with maximum-likelihood method (general time reversible plus gamma model with 7 discrete Gamma categories; 1,000 bootstrap replications). Values at the branches are bootstrap values of the corresponding neighbor-joining tree (maximum composite likelihood method); values <50% are not shown. Scale bar indicates an evolutionary distance of given substitutions per position in the sequence. B) Nucleocapsid open reading frame. C) Glycoprotein open reading frame. Scale bars indicate evolutionary distances of given substitutions per position in each sequence. LCMV, lymphocytic choriomeningitis virus.

Of the 812 rodents and shrews tested (Table 1), arenavirus RNA was found in lung tissue samples of 4 Namaqua rock mice (*Micaelamys* [*Aethomys*] *namaquensis*), 3 from Okahandja (N73, N80, N85) and 1 from Mariental (N27). Sanger sequencing of PCR products from a 338-nt fragment of the viral polymerase gene confirmed arenavirus-like origin. Initial phylogenetic analysis showed that the Okahandja specimens were related to MWV, but the sample from Mariental was a highly divergent member of the genus *Arenavirus* (Figure 2, panel A). Cell culture isolation was performed with samples N27 and N73 and resulted in 2 novel arenavirus isolates: Mariental virus (MRTV) and Okahandja virus (OKAV), respectively.

The genomes of the 2 arenaviruses were investigated by using next-generation sequencing and RT-PCR Sanger sequencing. The genome data obtained for MRTV and OKAV showed a typical arenavirus nucleotide composition with the L segment (MRTV: 6,840 nt; OKAV: 7,170 nt) coding for RNA polymerase and matrix protein and the S segment (MRTV: 3,360 nt; OKAV: 3,379 nt) coding for glycoprotein and nucleocapsid protein. Table 2 shows the nucleotide and amino acid sequence identities of nucleocapsid and glycoprotein open reading frame with other Old World (i.e., Eastern Hemisphere locations such as Europe, Asia, Africa) representatives of genus *Arenavirus*. On the basis of the nucleocapsid amino acid identity, OKAV is most related to MWV (75.7% identity). Furthermore, MRTV has the highest amino acid identity with IPPYV (71.4% identity) and with Gbagroube, Lassa, Luna, and Mobala viruses (≈70% identity).

**Table 2:** Nucleotide and amino acid identities of Mariental (MRTV) and Okahandja (OKAV) viruses compared with Old World representatives of the genus Arenavirus*

Virus species	S segment GenBank accession no.	GPC			NP	
		nt	aa		nt	aa
MRTV (N27)†	KM272987					
OKAV (N73)‡	KM272988	64.6	68.9		64.9	66.1
Gbagroube	GU830848	66.7	73.6		64.1	69.9
Ippy	NC_007905	66.4	**73.7**		64.1	**71.4**
Lassa	AY628203	67.4	73.2		65.5	69.8
LCMV	AB261991	57.4	57.2		61.1	63.6
Lujo	JX017360	47.7	38.2		60.1	56.7
Luna	AB586646	66.4	73.2		64.2	69.5
Lunk	NC_018710	57.4	54.1		61.2	62.5
Menekre	GU830862	66.5	72.3		65.1	68.3
Merino walk	GU078660	63.8	70.2		64.9	67.3
Mobala	NC_007903	63.8	72.1		64.6	70.5
OKAV (N73)‡	*KM272988*					
Gbagroube	GU830848	62.0	66.3		61.1	65.7
Ippy	NC_007905	62.9	69.4		62.3	66.2
Lassa	AY628203	64.6	68.2		60.8	65.9
LCMV	AB261991	58.9	57.1		62.0	63.6
Lujo	JX017360	47.6	38.4		60.0	57.8
Luna	AB586646	62.5	67.1		63.0	67.2
Lunk	NC_018710	56.1	55.1		60.6	62.6
Menekre	GU830862	63.7	70.4		62.9	65.9
Merino walk	GU078660	64.7	**76.1**		68.2	**75.7**
Mobala	NC_007903	62.0	66.5		63.6	64.5

*Identities are shown for glycoprotein and nucleocapsid open reading frames. Highest aa identity values are shown in **boldface**. S, small; GPC, glycoprotein; NP, nucleocapsid protein; nt, nucleotide; aa, amino acid; LCMV, lymphocytic choriomeningitis virus; L, large segment.  †Genome composition of MRTV (N27): (Z: 69–371; RdRP: 6,820–473; GPC: 49–1,527; NP: 3,297–1,710). Accession numbers for MRTV (N27) virus sequences: L, KP867641; S, KM272987. ‡Genome composition of OKAV (N73): Z, 58–336; RdRP, 7,121–435; GPC, 51–1,553; NP, 3,315–1,627. Accession numbers for virus sequences for OKAV N73: L, KP867642; S, KM272988; for OKAV N80: L, KM234277; for OKAV N85: L, KM234278.

In the nucleocapsid-based phylogenetic tree, OKAV clusters with 100% bootstrap support with MWV detected in *Myotomys unisulcatus* rodents in South Africa (Figure 2, panel B), and MRTV forms a clade with IPPYV isolated from *Praomys* spp. in the Central African Republic. The bootstrap support of this monophyletic group of the tree lies at 56%. The analysis of the glycoprotein open reading frame (Figure 2, panel C) leads to a similar result; OKAV shares the most recent common ancestor with MWV, and MRTV clusters with IPPYV but with a weaker bootstrap support.

## Conclusions

We detected and isolated 2 novel arenaviruses in Namibia, OKAV and MRTV. OKAV clearly clustered in relationship with the MWV from southern Africa, but MRTV is a more divergent member of the Old World arenavirus clade. According to amino acid identity and phylogenetic analysis, MRTV was most closely related to IPPYV from the Central African Republic; however, the low bootstrap support precluded a stringent estimation of this closest relative.

These new strains comply with the arenavirus species definition (*14*) on the basis of amino acid differences in nucleocapsid of >12% (>20% for both viruses), presence of specific host species, and existence of laboratory isolates. These properties indicate that MRTV and OKAV represent distinct arenavirus species.

These 2 viruses were found in the same host species located within a radius of 300 km. MRTV was found in only 1 sample (of 266); OKAV was detected in samples from 3 animals. Although more unlikely for OKAV than for MRTV, the possibility of a spillover infection to *M. namaquensis* from a still unknown reservoir host cannot be ruled out for either virus.

The Namaqua rock mouse’s habitat includes the tree and shrub savannahs of Namibia and most parts of southern Africa, including Namibia, South Africa, Botswana, Zimbabwe, and parts of Mozambique (*15*). These locations imply the possible occurrence of MRTV or OKAV in larger regions of the continent. Cell culture isolates and genomic sequence data are the first prerequisites for evaluating the public health relevance of these new viruses. Our findings extend the knowledge of geographic distribution and genetic diversity of arenaviruses in Africa.
